# Biologic Therapy and Malignancy Risk in Psoriasis: A Retrospective Cohort Study

**DOI:** 10.1111/1346-8138.17950

**Published:** 2025-09-08

**Authors:** Saori Takamura, Souichiro Saito, Sora Sugai, Yuki Shiko, Takumi Yamaguchi, Yohei Kawasaki, Tomoo Fukuda

**Affiliations:** ^1^ Department of Dermatology Saitama Medical Center, Saitama Medical University Saitama Japan; ^2^ Department of Biostatistics Graduate School of Medicine, Saitama Medical University Saitama Japan

**Keywords:** biologic therapy, Cox regression analysis, interleukin‐17/23 inhibitors, Kaplan–Meier analysis, malignancy risk, propensity score analysis, psoriasis

## Abstract

The long‐term impact of biologic therapy on malignancy risk in patients with psoriasis remains unclear. Given the chronic nature of psoriasis and the increasing use of biologics, understanding their long‐term safety profile is crucial. This study aimed to compare the incidence of malignancy between patients receiving continuous biologic therapy and those treated with topical monotherapy. We retrospectively analyzed 446 patients with psoriasis treated at a single center between May 2010 and March 2023 (biologic therapy: *n* = 226; topical monotherapy: *n* = 220). Malignancy incidence was estimated using the Kaplan–Meier method. Multivariable Cox proportional hazards regression with propensity score adjustment was employed to control for confounding variables. Subgroup analysis was performed within the biologic‐treated cohort to identify additional malignancy risk factors. Malignancies occurred in 11.2% (50/446) of patients, with rates of 4.4% (10/226) in the biologic therapy group and 18.2% (40/220) in the topical therapy group (*p* < 0.0001). Kaplan–Meier analysis demonstrated a significantly lower cumulative incidence of malignancy in the biologic group (*p* = 0.0035, log‐rank test). Cox regression confirmed that biologic therapy was associated with a significantly reduced malignancy risk (hazard ratio [HR]: 0.316; 95% confidence interval [CI]: 0.119–0.841; *p* = 0.0211). Notably, subgroup analysis using Cox regression within the biologic‐treated cohort revealed that family history of malignancy was significantly associated with an increased risk of malignancy (HR: 9.01; 95% CI: 1.69–4.87; *p* < 0.05). In conclusion, biologic therapy was associated with a significantly lower risk of malignancy in patients with psoriasis compared to topical monotherapy. Furthermore, family history of malignancy may represent an independent risk factor for malignancy among biologic‐treated patients. These findings underscore the importance of individualized cancer risk assessment when selecting treatment strategies and highlight the need for further prospective studies.

## 1 Introduction

Psoriasis is a systemic inflammatory disorder associated with multiple comorbidities, including metabolic syndrome, metabolic dysfunction‐associated steatotic liver disease (MASLD), and cardiovascular disease [1, 2]. Several epidemiological studies have reported an elevated malignancy risk in patients with psoriasis [3, 4].

The severity of psoriasis has been shown to correlate with malignancy‐related mortality [5]. However, the precise relationship between psoriasis and malignancy remains unclear. Previous studies have identified an increased incidence of cancers, including upper aerodigestive tract, liver, lung, pancreas, and urinary tract cancers, as well as lymphoma, squamous cell carcinoma (SCC), and basal cell carcinoma (BCC) [6, 7]. Chronic inflammation in psoriasis may contribute to carcinogenesis through cytokine and chemokine production, DNA damage, and epigenetic alterations [8, 9]. Furthermore, smoking, alcohol consumption, older age at psoriasis onset, and psoriatic erythroderma have been identified as malignancy risk factors in these patients [10, 11].

The potential carcinogenic effects of psoriasis treatments have also been a concern. Phototherapy and immune‐modulating therapies, including methotrexate, cyclosporine, retinoids, and biologics, have been suggested as possible contributors to malignancy development [12, 13]. However, the long‐term malignancy risk associated with biologic therapy remains controversial due to the lack of comprehensive adjustments for confounding factors.

A meta‐analysis identified an elevated risk of non‐melanoma skin cancers (NMSC), particularly SCC, in psoriasis patients treated with tumor necrosis factor inhibitors (TNFi) compared to both the general U.S. population and rheumatoid arthritis patients receiving mTNFi [14]. In contrast, another well‐designed meta‐analysis found no significant association between cumulative biologic exposure and the risk of cancers other than NMSC in psoriasis patients [4]. A retrospective cohort study from Japan involving 141 psoriasis patients who received biologic therapy suggested a trend toward reduced malignancy risk, though statistical significance was not achieved [11]. More recently, a global population‐based retrospective cohort study reported that psoriasis patients treated with interleukin (IL)‐17 inhibitors (IL‐17i) or IL‐23 inhibitors (IL‐23i) had a lower risk of several malignancies compared with those treated with TNFi [15].

To date, no study has directly compared the malignancy risk in psoriasis patients treated with biologic therapy versus those treated with topical monotherapy. Therefore, this study aimed to evaluate the incidence of malignancies in psoriasis patients undergoing continuous biologic therapy compared with those treated with topical therapy alone. Additionally, we sought to provide insights into the overall malignancy burden in psoriasis patients to inform clinical decision‐making.

## 2 Subjects and Methods

### 2.1 Study Design and Ethics

This retrospective study was conducted at Saitama Medical Center, Japan, in accordance with the ethical principles of the Declaration of Helsinki (1975) and was approved by the Institutional Review Board (Approval ID: 2024020). An opt‐out approach was employed to ensure patient confidentiality and compliance with ethical guidelines.

### 2.2 Patient Population

A total of 446 adult patients with psoriasis who received either biologic monotherapy (*n* = 226) or topical monotherapy (*n* = 220) between May 2010 and March 2023 at our institution were included in this study. All diagnoses were confirmed by board‐certified dermatologists based on clinical and histopathological findings. Eligible patients were defined as those diagnosed with one of the following psoriasis subtypes—plaque psoriasis, psoriatic arthritis (PsA), generalized pustular psoriasis (GPP), psoriatic erythroderma (PE), or guttate psoriasis—and who had received continuous treatment for at least 1 year with either biologic monotherapy or topical monotherapy throughout the observation period. To minimize potential confounding and ensure appropriate comparison between treatment groups, the following exclusion criteria were applied: (1) patients with a prior or concurrent history of malignancy at treatment initiation (*n* = 16); (2) patients who received other systemic therapies (e.g., phototherapy, retinoids, cyclosporine, methotrexate, or apremilast) or combination treatments (*n* = 57); and (3) patients for whom a minimum of 1 year of follow‐up could not be confirmed (*n* = 67). As a result, 446 out of 586 initially identified cases were included in the final analysis. These inclusion and exclusion criteria are visually summarized in Figure S1. In addition, during routine pre‐treatment screening conducted prior to the initiation of biologic therapy—including chest imaging and laboratory tests—several patients were incidentally found to have previously undiagnosed malignancies. As a result, biologic therapy was not initiated in these individuals, and they were excluded from the present analysis. Specifically, five patients (approximately 1.1% of those initially considered for biologic therapy) were newly diagnosed with malignancies during the screening process, including colorectal cancer, lung cancer, prostate cancer, thyroid cancer, and malignant lymphoma (one case each). These cases were deemed clinically ineligible for biologic treatment and were therefore not included in the final study population. Disease severity was assessed using the Psoriasis Area and Severity Index (PASI). GPP and PsA were diagnosed in accordance with Japanese clinical guidelines, while PE was defined as skin involvement affecting more than 80% of the body surface area.

### 2.3 Data Collection

Baseline clinical data were obtained from electronic medical records at the time of diagnosis. Variables included age, sex, body mass index (BMI), disease duration, comorbidities, laboratory findings, smoking history, and family history of malignancy. For patients who developed malignancies, detailed treatment histories before and after diagnosis were recorded.

### 2.4 Biologic Therapy

Among the 226 patients receiving biologic therapy, IL‐17 inhibitors were the most commonly prescribed agents, including infliximab (5.3%), adalimumab (11.5%), certolizumab pegol (6.6%), ustekinumab (13.7%), guselkumab (10.2%), risankizumab (14.2%), secukinumab (15.5%), ixekizumab (17.7%), and brodalumab (5.3%).

### 2.5 Statistical Analysis

Continuous variables are presented as medians (range), and categorical variables as frequencies (percentages). Group differences were assessed using the Wilcoxon rank‐sum test for continuous variables and Fisher's exact test for categorical variables. The cumulative incidence of malignancy was estimated using the Kaplan–Meier method, and differences between groups were compared using the log‐rank test.

Propensity scores were calculated via multivariate logistic regression incorporating clinically relevant baseline characteristics, including age, sex, BMI, smoking history, diabetes, hypertension, dyslipidemia, psoriasis duration, and PASI score. Due to a substantial imbalance in the distribution of propensity scores between treatment groups, we did not apply matching or inverse probability weighting (IPW) as the main analytical approach, as these methods would have resulted in a marked reduction in sample size or unstable estimates due to extreme weights. Instead, the primary analysis was conducted using a multivariable Cox proportional hazards model in which the propensity score was entered as a covariate (covariate adjustment method).

In addition, a sensitivity analysis was performed using a multivariable Cox regression model that included each confounding factor as an individual covariate. The results of this alternative model were consistent with the main analysis (HR = 0.31, 95% CI: 0.103–0.904, *p* = 0.03214), confirming the robustness of the findings.

To identify clinical factors independently associated with malignancy in patients treated with biologic agents, an additional multivariable Cox proportional hazards model was constructed within the biologic therapy group. Covariates included age, age at onset of psoriasis, PASI score, sex, smoking history, family history of malignancy, obesity (BMI > 25), diabetes, and dyslipidemia. This subgroup analysis aimed to explore potential predictors of malignancy among patients receiving biologic treatment. All statistical tests were two‐sided, and a *p* < 0.05 was considered statistically significant. Statistical analyses were performed using JMP Pro version 16 (SAS Institute Inc., Cary, NC, USA) and SAS software version 9.4.

## 3 Results

### 3.1 Patient Characteristics

This study included 446 patients, 72.9% of whom were male, with a median age of 55.0 years (range: 18–93). The median PASI score was 4.8, and psoriasis vulgaris (77.3%) and PsA (16.8%) were the most common subtypes. Comorbidities included hypertension (25.1%), dyslipidemia (25.1%), obesity (16.6%), and metabolic syndrome (53.6%). Smoking history was reported in 35.9% of patients, and a family history of malignancy in 6.1%. Table 1 presents a summary of patient characteristics. Significant differences were observed between groups in age at onset, body weight, disease duration, PASI score, psoriasis subtype distribution, smoking history, and prevalence of dyslipidemia.

**TABLE 1:** Baseline characteristics of patients before propensity score adjustment.

Characteristic	Biologic therapy (*N* = 226)	Topical therapy (*N* = 220)	*p*
Sex (female/male)	23.9%/76.1%	30.5%/69.5%	0.119
Age (years)	55.0 (46.0–66.0)	56.0 (39.5–66.0)	0.173
Age at psoriasis onset (years)	35.5 (25.0–49.0)	50.0 (33.0–63.5)	< 0.0001
Body weight (kg)	70.0 (62.0–79.5)	68.0 (57.0–78.0)	0.032
Body mass index (kg/m^2^)	25.5 (22.6–28.6)	24.9 (22.4–28.7)	0.724
Psoriasis duration (years)	16.0 (9.0–25.0)	3.0 (1.0–8.0)	< 0.0001
Baseline psoriasis area and severity index	7.8 (3.6–13.8)	3.6 (2.0–5.6)	< 0.0001
Psoriasis vulgaris	58.0% (131)	97.2% (214)	< 0.0001
Psoriatic arthritis	32.3% (73)	0.9% (2)	< 0.0001
Generalized pustular psoriasis	4.9% (11)	0.5% (1)	0.006
Psoriatic erythroderma	4.4% (10)	0.5% (1)	0.004
Psoriasis guttata	0.0% (0)	0.9% (2)	0.317
History of smoking	58.0% (131)	13.2% (29)	< 0.0001
Family history of malignancy	7.1% (16)	5.0% (11)	0.239
Dyslipidemia	34.0% (77)	15.9% (35)	< 0.0001
Diabetes mellitus	19.0% (43)	14.1% (31)	0.161
Obesity	56.0% (124)	49.3% (69)	0.148

*Note:* Data are presented as median (interquartile range [IQR]), range, or number. Baseline characteristics of psoriasis patients receiving biologic therapy (*n* = 226) or topical therapy (*n* = 220). Continuous variables are expressed as medians with interquartile range (IQR) or range, while categorical variables are presented as percentages. Significant differences between the groups were observed in age at psoriasis onset, body weight, psoriasis duration, baseline Psoriasis Area and Severity Index (PASI), psoriasis subtype distribution, smoking history, and prevalence of dyslipidemia (*p* < 0.05).

### 3.2 Malignancy Incidence and Distribution

Over a mean follow‐up of 5.5 years, 50 patients (11.2%) developed malignancies. The median age at malignancy diagnosis was 66.3 years, with a mean interval of 15.5 ± 2.1 years between psoriasis onset and malignancy detection. Most patients (92.0%) developed a single malignancy, while 8.0% had multiple malignancies. The most common malignancies were colorectal cancer (23.6%), liver/bile duct cancer (12.7%), and prostate cancer (12.7%) (Table 2). The median observation period from treatment initiation to the end of follow‐up was 4 years (range: 1–13 years) in the biologic therapy group and 5 years (range: 1–13 years) in the topical therapy group. A statistical comparison of observation durations between the two groups revealed no significant difference, indicating that malignancy risk was evaluated over comparable timeframes.

**TABLE 2:** Malignancy types and frequencies.

Malignancy type	Number of cases (*n*)	Frequency (%)
Colorectal cancer	13	23.6
Liver/bile duct cancer	7	12.7
Prostate cancer	7	12.7
Breast cancer	5	9.1
Gastric cancer	5	9.1
Bladder cancer	3	5.5
Malignant lymphoma	3	5.5
Renal cancer	3	5.5
Lung cancer	2	3.6
Thyroid cancer	1	1.8
Head and neck cancer	1	1.8
Uterine cancer	1	1.8
Ovarian cancer	1	1.8
Skin cancer	1	1.8
Pancreatic cancer	1	1.8
Duodenal adenocarcinoma	1	1.8

*Note:* Data are presented as the number of cases and percentage of total malignancies. This table summarizes the types and frequencies of malignancies observed in patients with psoriasis. Colorectal cancer was the most prevalent malignancy, accounting for 23.6% of cases, followed by liver/bile duct cancer and prostate cancer, each representing 12.7%. A total of 55 malignancies were identified in the cohort.

Abbreviation: *n*, number of cases.

### 3.3 Biologic Therapy and Malignancy Risk

**FIGURE 1:**
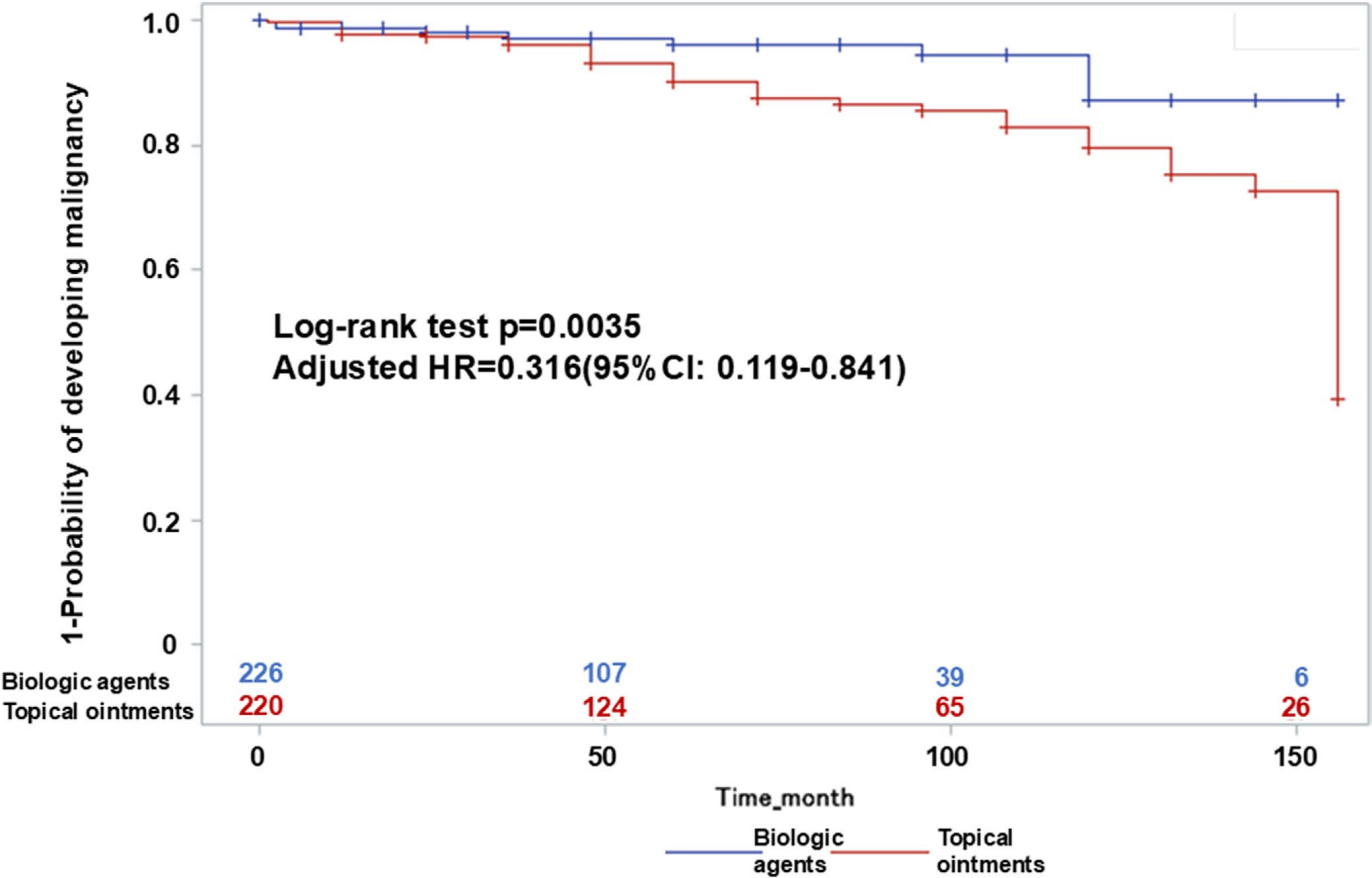
Cumulative incidence of malignancies in psoriasis patients treated with biologic therapy vs. topical therapy. The cumulative incidence of malignancies in patients with psoriasis receiving biologic therapy or topical therapy was estimated using the Kaplan–Meier method. Differences between the groups were evaluated using the log‐rank test (*p* = 0.0035). Data were analyzed using the Kaplan–Meier method to estimate malignancy incidence over time, with statistical significance determined by the log‐rank test. Abbreviation: *p*, *p*‐value.

The annual incidence of malignancy was significantly lower in the biologic therapy group (4.4%) compared to the topical monotherapy group (18.2%) (*p* < 0.0001). Kaplan–Meier analysis revealed a significantly reduced cumulative incidence of malignancy in patients treated with biologics compared to those receiving topical therapy (*p* = 0.0035, log‐rank test) (Figure 1). These findings suggest a potential protective effect of biologic agents against malignancy development in patients with psoriasis.

### 3.4 Multivariate Analysis of Malignancy Risk

To account for baseline differences between the treatment groups, we calculated propensity scores using multivariate logistic regression based on age, sex, BMI, smoking history, diabetes, hypertension, dyslipidemia, psoriasis duration, and PASI score. A multivariable Cox proportional hazards model incorporating the propensity score as a covariate was used as the primary analysis. This model demonstrated that biologic therapy was significantly associated with a lower risk of malignancy compared with topical monotherapy (HR = 0.316; 95% CI: 0.119–0.841; *p* = 0.0211).

To confirm the robustness of this association, a sensitivity analysis was conducted using an alternative multivariable Cox model in which each confounding factor was entered as an independent covariate rather than using the propensity score. This analysis yielded consistent results (HR = 0.31; 95% CI: 0.103–0.904; *p* = 0.03214), reinforcing the observed inverse relationship between biologic therapy and malignancy risk.

### 3.5 Subgroup Analysis of Malignancy Risk Factors in Biologic‐Treated Patients

To investigate clinical factors associated with malignancy development among patients treated with biologic agents, we conducted a multivariable Cox proportional hazards analysis within the biologic therapy group (Table 3). Patients who developed malignancies during the observation period were compared with those who did not. Covariates included sex, age, baseline PASI, smoking history, family history of malignancy, diabetes mellitus, and obesity (defined as BMI > 25). Among these variables, only a family history of malignancy was significantly associated with an increased risk of cancer development (HR = 9.01; 95% CI: 1.69– 4.87; *p* = 0.017). No other factors demonstrated statistically significant associations. These findings suggest that a familial or inherited predisposition to cancer may remain an important risk factor for malignancy even in patients receiving intensive immunomodulatory therapy. However, given the relatively small number of malignancy events in this subgroup, the results should be interpreted with caution. Larger prospective studies are warranted to validate this association and further explore potential gene–environment interactions in biologic‐treated psoriasis populations.

**TABLE 3:** Multivariate cox regression analysis of malignancy risk factors in psoriasis patients treated with biologic agents.

	Adjusted model	
	HR (95% CI)	*p*
Sex (male)	1.32 (0.26–6.67)	0.736
Age	0.97 (0.92–1.02)	0.329
Baseline PASI	0.99 (0.93–1.07)	0.771
History of smoking	1.59 (0.35–7.14)	0.543
Family history of malignancy	9.01 (1.69–4.87)	0.017
Diabetes mellitus	1.71 (0.37–7.84)	0.503
Obesity	0.97 (0.24–3.97)	0.971

*Note:* Obesity was defined as BMI > 25 kg/m^2^. This table presents the results of a multivariate Cox proportional hazards regression analysis assessing the association between clinical variables and the incidence of malignancy in psoriasis patients receiving biologic therapy. Hazard ratios (HRs), 95% confidence intervals (CIs), and *p*‐values are shown for each variable. Significant association was observed for age at onset of psoriasis.

Abbreviations: CI, confidence interval; HR, hazard ratio; PASI, psoriasis area and severity index.

## 4 Discussion

This study demonstrated that the annual incidence of malignancy was significantly lower in psoriasis patients treated with biologic agents compared to those receiving topical monotherapy. Even after adjusting for potential confounders using a multivariable Cox proportional hazards model incorporating propensity scores, biologic therapy remained significantly associated with a reduced risk of malignancy. Additionally, colorectal cancer was the most frequently observed malignancy, followed by liver/bile duct and prostate cancers. Importantly, an exploratory multivariable analysis within the biologic‐treated group revealed that family history of malignancy was significantly associated with an increased risk of malignancy. This novel finding suggests that a later onset of psoriasis may reflect underlying immunosenescence or an age‐related increase in cancer susceptibility. It provides a clinically meaningful perspective for risk stratification when initiating biologic therapy, particularly in older patients. Further research is warranted to elucidate the mechanisms linking family history of malignancy with cancer development and to guide personalized cancer surveillance strategies in this population.

In a previous retrospective cohort study conducted in Japan, the prevalence of colorectal and liver/bile duct cancers among psoriasis patients was reported to be 20.9% and 6.0%, respectively [11]. In our cohort, these rates were slightly higher, at 23.6% and 12.7%, respectively. This discrepancy may be partially explained by the inclusion of patients with metabolic dysfunction‐associated steatotic liver disease (MASLD), as suggested in our earlier reports [2]. MASLD has been implicated in the pathogenesis of both colorectal cancer and hepatocellular carcinoma (HCC) [16, 17]. Moreover, recent systematic reviews from the United States, Sweden, Switzerland, Denmark, and Taiwan have demonstrated an increased risk of colorectal cancer in patients with psoriasis compared to the general population [18]. These findings indicate that our study population shares similar risk characteristics with those reported in prior international research.

Our study further revealed that biologic therapy significantly reduced the annual malignancy incidence compared to topical monotherapy. This finding was supported by multivariate analysis using propensity scores as covariates. A prior study by Watanabe et al. observed a trend toward reduced malignancy risk in psoriasis patients treated with biologic agents, although statistical significance was not achieved [11]. The stronger effect observed in our study may be attributed to our exclusion of patients receiving phototherapy or immune‐modulating therapies, focusing solely on biologic versus topical treatments. Moreover, a global population‐based retrospective cohort study found that IL‐17i and IL‐23i were associated with a reduced malignancy risk compared to TNFi [15]. These findings align with our results, suggesting that psoriasis and malignancies may share common IL‐17‐ and IL‐23‐mediated pathways.

IL‐17 is a pivotal cytokine in psoriasis pathogenesis [19] and has been implicated in tumor development at various stages [20]. Experimental models have shown that IL‐17 ablation reduces cancer progression in organs such as the colon, pancreas, liver, skin, and lungs. Elevated levels of IL‐17A‐producing cells in colon cancer patients have been associated with poor prognosis, potentially due to vascular endothelial growth factor expression [21]. In HCC, the density of intratumoral IL‐17‐producing cells, primarily Th17 cells, has been shown to be significantly higher than in adjacent non‐tumorous tissues. This accumulation is associated with increased microvessel density and poorer prognosis [22]. Furthermore, IL‐17A and Th17 cells have been implicated in the progression of non‐alcoholic steatohepatitis (NASH) to HCC, highlighting their role in tumor angiogenesis and inflammation‐driven carcinogenesis [23]. IL‐23 has also been shown to promote tumor growth, as mice lacking IL‐23p19 exhibit resistance to certain tumor models [24]. These mechanistic insights support our findings that IL‐17i and IL‐23i may offer protective effects against tumor development in patients with psoriasis.

This study has several limitations. First, it was conducted at a single center with a limited sample size, which may restrict the generalizability of our findings and reduce the statistical power to evaluate malignancy risk for individual biologic agents. Second, data on alcohol consumption during treatment were unavailable, potentially confounding the observed associations. Third, selection bias may have influenced the malignancy incidence observed in the biologic therapy group. All patients considered for biologic treatment underwent routine pre‐treatment screening for infectious diseases, including chest imaging and blood tests. During this process, five patients (1.1%) were incidentally diagnosed with previously undetected malignancies—specifically colorectal, lung, prostate, and thyroid cancers, as well as malignant lymphoma. These individuals were excluded from the biologic therapy cohort and the final analysis. Consequently, the biologic group may have been enriched for healthier individuals, potentially leading to an underestimation of malignancy risk in this cohort. Fourth, follow‐up intensity differed between treatment groups. Patients receiving biologic therapy were generally monitored at 3‐month intervals, including regular blood tests, whereas those treated with topical monotherapy were often seen less frequently (e.g., every 6 months–1 year if clinically stable). This discrepancy in clinical surveillance may have affected malignancy detection rates, thereby introducing a potential detection bias. Finally, although we adjusted for major clinical confounders using a multivariable Cox regression model incorporating propensity scores, the possibility of residual or unmeasured confounding cannot be fully excluded. Future multicenter, prospective studies incorporating standardized screening protocols, uniform follow‐up intervals, and comprehensive lifestyle data—including alcohol consumption and physical activity—are needed to validate these findings and improve external validity.

In conclusion, biologic therapy was associated with a significantly reduced risk of malignancy in patients with psoriasis, even after adjusting for key clinical confounders. Additionally, our subgroup analysis within the biologic‐treated cohort revealed that a family history of malignancy was independently associated with increased cancer risk. These findings highlight the importance of individualized risk assessment when selecting treatment strategies and suggest that biologic therapy may serve as a viable and potentially safer option for patients with an elevated baseline cancer risk. Nonetheless, further large‐scale, prospective studies are warranted to validate these observations and to inform personalized, risk‐adapted management approaches in clinical practice.

## Conflicts of Interest

S.T. has received lecture fees from Torii Pharmaceutical, Takeda Pharmaceutical, CSL Behring, AbbVie, UCB Japan, Janssen Pharmaceutical, Taiho Pharmaceutical, Maruho, Novartis Pharma, Kyowa Hakko Kirin, Eli Lilly, LEO Pharma, and Sanofi. T.F. has received lecture fees from Sato Pharmaceutical, Eli Lilly, AbbVie, and CSL Behring. Drs. Saito, Sugai, Shiko, Yamaguchi, and Kawasaki declare no conflicts of interest.

## Supporting information


**Figure S1:** Flowchart of patient selection. A total of 586 potentially eligible psoriasis patients were identified in the institutional database. Of these, 16 patients with a current or past diagnosis of malignancy at treatment initiation were excluded. An additional 57 patients were excluded due to the use of other systemic therapies (e.g., phototherapy, retinoids, cyclosporine, methotrexate, or apremilast) or combination treatment regimens. Furthermore, 67 patients were excluded because they did not meet the minimum requirement of 1 year of continuous treatment with biologic monotherapy or topical monotherapy. As a result, 446 patients were included in the final analysis.

## Data Availability

The data that support the findings of this study are available on request from the corresponding author. The data are not publicly available due to privacy or ethical restrictions.
