# Supplementary figures and images for: Biologic Therapy and Malignancy Risk in Psoriasis: A Retrospective Cohort Study
Source: J Dermatol. 2025 Sep 8;52(12):1796–802. doi: 10.1111/1346-8138.17950 (PMC12698904; doi:10.1111/1346-8138.17950)

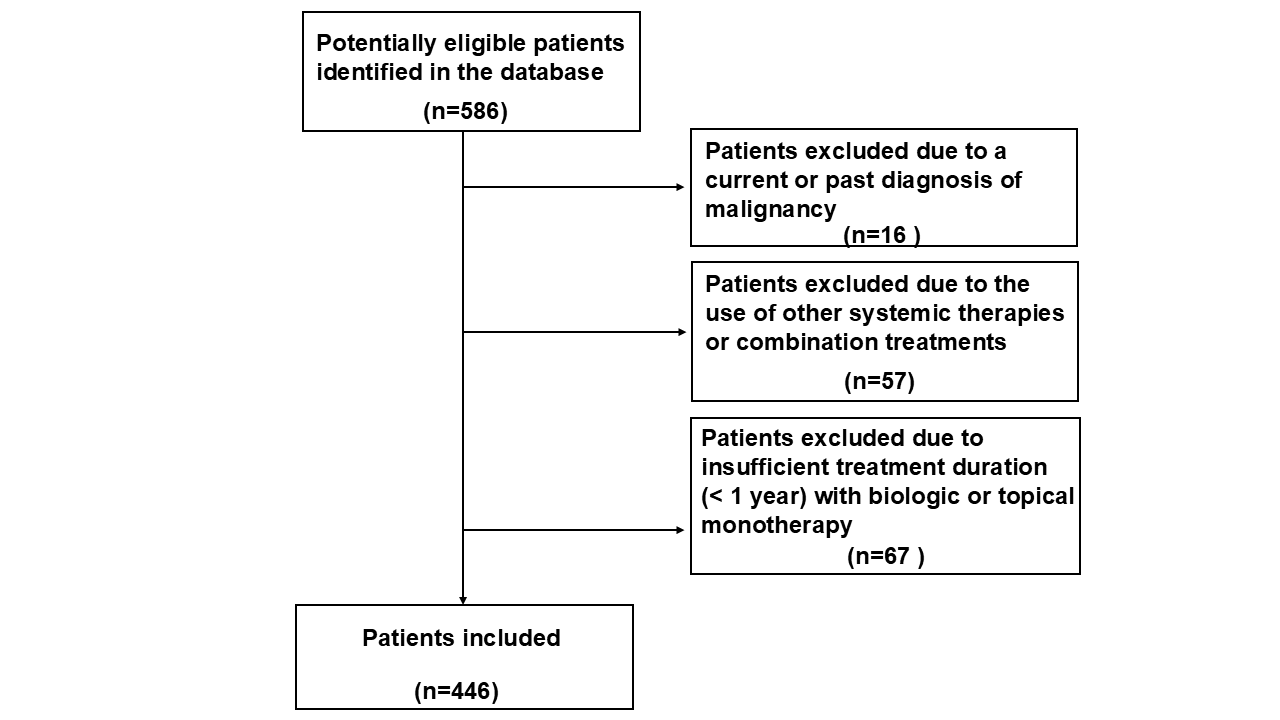

Supplement: Supplementary file 1 — Figure S1: Flowchart of patient selection. A total of 586 potentially eligible psoriasis patients were identified in the institutional database. Of these, 16 patients with a current or past diagnosis of malignancy at treatment initiation were excluded. An additional 57 patients were excluded due to the use of other systemic therapies (e.g., phototherapy, retinoids, cyclosporine, methotrexate, or apremilast) or combination treatment regimens. Furthermore, 67 patients were excluded because they did not meet the minimum requirement of 1 year of continuous treatment with biologic monotherapy or topical monotherapy. As a result, 446 patients were included in the final analysis. [file JDE-52-1796-s001.tif]
